# Strategies Used by Emergency Department Clinical Champions to Sustain Improvements in Intimate Partner Violence Screening: A Longitudinal Qualitative Study

**DOI:** 10.1155/jonm/6615231

**Published:** 2025-05-18

**Authors:** Elham Saberi, John Hurley, Marie Hutchinson

**Affiliations:** ^1^School of Health and Human Sciences, Southern Cross University, P.O. Box 157, Lismore, New South Wales, Australia; ^2^School of Health and Human Sciences, Southern Cross University, Coffs Harbour, Australia

**Keywords:** champion, change agent, domestic violence, emergency department, intimate partner violence, quality improvement, screening

## Abstract

**Aim:** To identify the strategies that clinical champions implemented within emergency departments to initiate and sustain routine intimate partner violence (IPV) screening.

**Background:** For effective IPV identification and responses to occur within healthcare settings, new attitudinal and practice changes are required. This paper adds to the body of knowledge about champions and their role within healthcare settings to achieve this end.

**Design:** This qualitative study involved semistructured interviews with 23 individuals over a 2-year period who identified as champions and worked to introduce routine IPV screening in two hospital emergency departments. Data from transcribed interviews were analysed thematically using an interpretive framework and a process of constant comparison.

**Results:** Champions detailed a range of strategies they employed to foster practice change. The primary strategies were as follows: normalising enquiring about IPV through narrative practice, building understanding and ownership, providing accompaniment, serving as an educator and resource person, and managing resistance.

**Conclusions and Implications:** This paper highlights the specific complexities champions face when bringing about practice change in the area of IPV. The findings suggest nurse champions, particularly those working in the IPV space, adopt unique strategies compared to those described in the mainstream literature on champions. This evidence has implications for best practice and can ensure that the champion role is more effectively utilised by health services to better meet the needs of individuals experiencing IPV. It is recommended that champions be established as part of any IPV practice change process. Introduction of IPV education into nursing undergraduate and postgraduate education and workplace ongoing education and training is also recommended to strengthen the capacity of nursing staff to serve as IPV practice change champions.

## 1. Introduction

Intimate partner violence (IPV) is recognised as a significant global health issue, with nearly 30% of women reporting experience of some form of IPV in their lifetime [1]. In Australia, it is estimated that 27% of all women experience violence by a partner after the age of 15 and 23% experience emotional abuse [2]. In heterosexual relationships, women more frequently experience IPV, and it is often more severe [1, 3]. There is also growing recognition that IPV affects people of all genders, leading to increased attention on the experiences of men and noncisgender women [4, 5]. Individuals experiencing IPV access health services more frequently, particularly emergency departments (EDs) [6, 7]. Therefore, frontline nurses, who provide direct service to patients in the EDs, play an important role in identifying and responding to the care needs of these individuals [8, 9].

There are unique organisational challenges in the implementation of IPV enquiry and responses in healthcare. This can encompass high patient volumes, limited time due to competing demands, poor integration with existing processes and electronic medical records and a lack of privacy in busy settings like the ED [10]. Even though evidence shows that between 2% and 4.3% of women experience injuries caused by IPV in the year before attending EDs [11], research confirms much of this abuse goes undetected when women are presented to hospitals [12]. Routine inquiry about IPV has been reported to increase identification of those at risk by 133% when compared with usual care [13]. In America, routine screening for IPV is utilised in EDs and is recommended by the American College of Emergency Physicians [14]. In Australia, routine screening is not mandated in EDs and is not usual practice for ED nurses. In NSW, however, screening for IPV within EDs has been piloted and found to be feasible [15]. Although men also experience this violence, there is currently insufficient evidence regarding IPV screening for men. Given this evidence base, routine screening is not widely recommended [16], and it is not recommended in the setting where this study was conducted. In order for routine IPV screening to be implemented and sustained, a change in nurses' perceptions of their responsibilities and a more holistic approach towards patients is required [9].

Evidence suggests that routine screening for IPV is not easy to sustain, even in areas of the health system where it is mandated as routine practice [17]. Nurses working in EDs are reported to feel uncomfortable and ill-prepared to enquire about and respond to IPV [18]. Many nurses are unaware of policies and protocols and the screening tools that are utilised within their service [7]. For some nurses, identification and response to IPV is viewed as a personal choice rather than a professional responsibility that is within their scope of practice [9], and lack of time and privacy have also been raised as barriers to screening for IPV within EDs [10]. Others report that ED nurses are willing to screen and believe that it is important [19].

Recent evidence shows a connection between the confidence of nurses to screen and respond to IPV and the training and preparation they have received [20, 21]. Working within a team who are highly dedicated to addressing IPV, being supported in the workplace, collaborating with and receiving support from specialist IPV nursing staff and receiving adequate training and preparation, particularly training focussed on the duty to screen, increases clinician confidence and readiness to address IPV within clinical settings [7, 19–22]. This evidence suggests that individuals who are identified as champions who are committed and able to support staff to change their practice would be invaluable in bringing about improved responses to IPV across healthcare settings.

Champions are defined as individuals who emerge either formally or informally [23] and contribute to change or innovation by building support, overcoming resistance and ensuring changes are implemented [24]. In healthcare settings, research evidence confirms champions' support innovation and plays an important role in the uptake of quality improvement and evidence-based practice [25, 26]. A recent scoping review highlighted a paucity of research evidence on champions in the field of IPV [24]. Evidence on the role champions' play in mentoring and supporting healthcare workers to change their IPV practice is required for clinical leaders to be able to effectively identify, prepare, engage and sustain champions to bring about practice change within their settings and to better meet the needs of individuals accessing their services who are experiencing IPV.

This is the first study to investigate the experience of IPV practice change champions. The champions worked in two major regional tertiary hospital EDs to introduce and embed IPV routine screening of women. The study explored the characteristics, motivations and strategies of nurse champions who promoted routine IPV screening, examining the barriers and enablers they encountered in establishing and sustaining this practice. This paper reports on the strategies used by champions to bring about system and practice change and how these individuals impacted IPV routine screening. It provides novel insights into the role of champions in bringing about practice change within nurse practice settings to meet the needs of patients experiencing IPV.

## 2. Method

### 2.1. Design

This qualitative study took place between 2017 and 2019. Longitudinal qualitative semistructured interviews were conducted over this period with 23 individuals who identified as ED champions of IPV practice change. The longitudinal design was chosen to ensure that the rich experience of champions would be captured over the 2-year period, whilst IPV routine screening was implemented and embedded into routine practice. Longitudinal studies allow for data to be collected over a period of time either from the same participants or from different participants within the same setting [27–30]. In this study, different participants from the same settings were interviewed over the 2 year period. The serial nature of the interviews allowed for interviews with different types of champions and offered insights into the evolving dynamics and experiences over time at two different sites. The interviews were digitally audio-recorded (with permission) and transcribed verbatim. As all champions in the study were interviewed, theoretical saturation was reached when the analysis was no longer yielding new insights, no new themes could be identified, and no new questions or gaps in information were identified [31]. At this point, it was felt that all the concepts were well defined and explained, and the breadth and depth of categories and their relationship to each other had been adequately explored for the study [31, 32].

This study used the theoretical lens of social constructionism to shape the research design, data collection and data analysis. Social constructionism is a theoretical perspective that emphasises social relationships as the focus of generating truths and realities and attributes knowledge to social processes in creating shared understandings of realities [33]. The hermeneutic circle was applied to data analysis to provide deeper understanding over time as patterns emerged from the analysis of interview data and through the mingling of ideas and experiences of the researcher and the participants. In hermeneutics, interpretation is seen as a subjective and dynamic process influenced by various factors such as language, culture and personal experiences [34]. The fundamental principle of the hermeneutic circle is the need to understand the phenomenon from different perspectives and to give meaning to the whole from its multiple parts and its parts from the whole [34].

### 2.2. Sample

Participants were recruited from two major regional tertiary hospitals in New South Wales, Australia.  Table 1 summarises the demographic characteristics of the participants in the study.

In 2017, before the implementation of this study, a 6-month feasibility study was conducted in a number of sites across the state to establish the feasibility and acceptability of screening for IPV in the ED. Clinicians involved in the feasibility study reported high levels of acceptability (91%), and nearly all agreed that screening was appropriate (87%) [10]. One ED within the Local Health District (Site 1) was involved with this feasibility study and as such some of the interviews carried out with champions at Site 1 were conducted in 2017. In 2018, the IPV routine screening process was reintroduced in Site 1. One year later, in 2019, the screening process was introduced into a second ED (Site 2). The screening implemented was undertaken with all women aged 16 years or greater. Nurses implemented the screening as part of their regular practice using the HITS screening tool [35]. The name HITS is made up of the first letters of the four validated Likert scale questions in the tool. These questions were embedded into existing assessment processes, and screening only occurred if the woman was alone. The questions asked women how often their partner had hurt, insulted, threatened or screamed/sworn at them in the previous year. In each question, women were asked to provide a score from 1 to 5 (never to frequently) according to frequency of the abuse for each item. Possible scores could range from 4–20, and a score of 10 or greater was considered indicative of IPV [15]. When IPV was identified, women were offered psychosocial support through a referral to hospital social work and crisis care and follow-up case management through referral to a local nongovernment specialist IPV service. The feasibility and acceptability of this brief screening in the ED was established through an earlier pilot study [15].

During the project establishment period champions were identified at both sites, and by 2019, a team of champions were in place at each site and at various levels of management. Champions supported the implementation and sustainability of IPV screening uptake. Frontline nursing champions (reported as ‘Nurse Champions' herein) supported the practice change on a day-to-day basis in the clinical setting. These champions were either nominated by their manager or self-nominated through an expression of interest process. All nursing staff in the two EDs were invited to nominate as champions, and all individuals who nominated were accepted as champions and none declined or were unable to participate. Manager champions (reported as ‘Manager Champions') and executive champions (reported as ‘Executive Champions') included service level managers and managers that were at the director and executive levels of the organisation. They focussed on ensuring that the change process was implemented effectively and driven and supported at the service and higher levels of the organisation.

### 2.3. Data Collection

All individuals who served as champions to support the implementation and sustaining of IPV screening were recruited as the study participants (see Table 1).

Semistructured interviews were conducted at two stages of implementing the routine IPV screening project in two EDs. In 2017, seven interviews were conducted with champions at Site 1 and with 2 champions who had a district-wide role. Subsequently, in 2019, 14 interviews were conducted with additional champions from Site 1 and champions from Site 2. All interviews were conducted by the same researcher.

An interview guide based on the research aims was developed. It included questions regarding individuals' role in the implementation of IPV screening, their motivation, capabilities, ways of working and strategies in championing IPV screening. To gain deeper understanding, participants were prompted to provide more information or reflect in greater detail on their experiences using prompts such as ‘Can you tell me a little bit more about that', ‘Can you explain that to me', ‘How did that feel' or ‘How did that work'. The longitudinal design of the study allowed for the adaptation of probes as insights emerged. Interviews were held face-to-face in a private area at the workplace of the participants and lasted approximately one and a half hours. Written consent was acquired from all participants.

### 2.4. Data Analysis

Interviews were transcribed verbatim. The researcher deidentified the transcripts by replacing names with pseudonyms and adapting or redacting details in events, locations, genders or dates to ensure the location of the event or the identity of participants was not identifiable. After transcription, participants were provided with a copy of their interview transcripts for review and verification. Following immersion in the data, sections of transcripts that were salient or captured the essence meaning were coded through a process of constant comparison [36]. A new code was applied each time a new topic was identified, and the same code was used when the topics were similar. As the coding emerged, similar or comparable codes were grouped to form clusters, which were refined through a repeating process of collation and refinement to form themes. Several strategies were employed to establish transparency, credibility and trustworthiness during the analysis. Initially, the three team members undertook individual inductive coding of a subset of the interviews and compared emerging interpretations to derive a shared understanding. The thematic analysis carried out complied with the criteria set out by Lincoln and Guba [37, 38]. The final stage of collaborative analysis involved the team members discussing and revising the overarching narrative and identifying key elements of the emerging themes and subthemes [39]. In line with the hermeneutic approach to the analysis and interpretation, this analytic triangulation required the team members to adopt reflexivity when attempting to reach consensus. The manual coding was undertaken in Microsoft Office using colour highlight and text comments to tag text, followed by sorting into tables [40]. Three main themes were derived from the data. The theme *champion strategies* is reported here. The term domestic violence (DV) appears in some participant narratives. This reflects the use of the term in Australia, where DV is used interchangeably with the term IPV. The language of study participants reflects their localised contexts.

### 2.5. Ethical Considerations

Ethics approval was sought and gained from the local health district and university's Human Research Ethics Committees. Prior to being enrolled into the study, participants were provided with written information about the study and each provided written consent.

## 3. Results

### 3.1. Sample Characteristics

The final sample consisted of 23 participants. All participants were employed in the two hospitals and self-identified as champions of the IPV practice change occurring in the EDs. The first few champions recruited at Site 1 were identified by their manager. All champions after that time self-identified through an expression of interest process. Manager and executive champions self-identified without an expression of interest, as they engaged with the project.  Table 1 summarises the characteristics of the study participants.

### 3.2. Champion Strategies

In the interview, champions were asked to describe the strategies they employed to bring about practice change. Most described using some or all of the following five strategies: normalising enquiring about IPV through narrative practice, building understanding and ownership, providing accompaniment, serving as an educator and resource person and managing resistance.

#### 3.2.1. Normalising Enquiring About IPV Through Narrative Practice

Common among champions was the use of narrative practice to construct broader ownership of enquiring about IPV as routine practice.

Moving the conversation from one of whether or not to screen to one of how to make screening ‘*just a normal part of the general screening process' (Participant 15, Manager Champion).*

In opening up conversation, champions aimed to ensure clinicians in the ED were speaking more openly about IPV and taking ownership of acting on the issue; rather than viewing screening and inquiry as someone else's role. Champions shared that by continually talking about IPV, it became a part of the language of the ED:

*‘If we could all get into a place where we could work IPV screening into our handovers and into our lingo, continuously talking about it, I think that would have a really good effect' (Participant 3, Nurse Champion).*

This strategy served to normalise the topic of IPV within the ED, allowing it to be openly spoken about, with both patients and colleagues. One manager champion shared that the aim was

*‘At any point in the interaction of a patient with the service providers, to consider this issue so that they (the patient) can disclose. They might initially refuse … So, there'd be an opportunity at repeated episodes during the continuum of care to reconsider their preparedness to participate … It would be ideal if we could get into a situation where it was' (Participant 15, Manager Champion).*

Storytelling was an important component of narrative practice and was used by champions to share positive outcomes for women resulting from the screening process:

‘I tell them stories … every time I get a positive story' (Participant 6, Nurse Champion). Narratives such as; ‘We looked after a patient the other day, we did a HITS screen, off the charts, totally unexpected' (Participant 11, Nurse Champion) and; ‘Did you know on day one, a nurse asked these questions and she was really surprised' (Participant 22, Nurse Champion) raised awareness among staff about patient outcomes from screening.

Champion efforts through conversations and strengthening relationships reminded staff of the importance of screening, normalised the topic of IPV, and built staff confidence, engagement and ownership for the process. As one participant shared:

*‘… unless we talk about it, we forget about it, so we need to constantly talk about it.… The more we talk about it the more familiar it becomes and the more people are happy to ask questions' (Participant 22, Nurse Champion).*

Open narratives were also important in ‘*taking the taboo away' (Participant 9, Nurse Champion).* In opening narratives about taboos, champion participants understood that the topic of IPV was ‘very *confronting for a lot of the staff … as they don't know how to react to it'* (*Participant 6,* Nurse Champion). Over time, IPV became a topic that was more comfortably spoken about and part of everyday practice: *‘People kind of see it as a process now rather than “Oh yeah that's what we've got to do” and we've talked about it now–it's like “Oh yeah, we've got to do their Obs and we've got to do these screening tools”' (Participant 8, Nurse Champion).*

#### 3.2.2. Building Understanding and Ownership

A key strategy utilised by champions was raising staff awareness about why IPV and IPV screening were important and why screening made a difference to patient outcomes. Given the complexities of IPV, participants reported the need to be particularly sensitive and cater their engagement strategies to the need of each staff member and what motivated this staff member to enquire about IPV.

In building ownership, champions sought to motivate clinicians to act from a place of compassion and a desire to make a difference rather than knowledge or complying with a directive. As one participant explained, *‘You can't tell nurses to do it. You have to take the approach for them to want to do it' (Participant 6, Nurse Champion).* And *‘I think people need to feel special and people need to feel that they're doing it for the right reason' (Participant 5, Nurse Champion).*

One of the manager participants shared that they found it *‘interesting as a manager trying to understand what motivates individuals within the workforce.'* This participant went on to say that once she understood the staff member's motivation style, she tailored, *‘the education and information to that motivation style to get the staff member on board' (Participant 16, Manager Champion).*

Participants also spoke about being mindful of the individual barriers that may have stopped staff from changing their practice. One participant shared:

*‘…you can't just tell them to do it. You've got to get them to understand why they should do it. You've got to help them break down the barriers of why they can't do it. And everyone's different so you've got to be very sensitive to each person… Everyone's had their own personal experience from domestic violence' (Participant 6, Nurse Champion).*

Another aspect of building understanding and ownership was a nurturing environment for staff that affirmed their practice, rather than focussing on challenges. Affirmations were emphasised as an important part of the transformational dialogue champions utilised to bring about change in practice:

*‘I think it's really important to recognise when staff are doing it. A pat on the back is something we don't do a lot of in Health. We all need to be recognised … You need to see that you're making a difference. You know you're doing all this work you need to see the outcomes' (Participant 9, Nurse Champion).*

#### 3.2.3. Providing Accompaniment

Champions also described creating an environment of learning and reflection through accompaniment that connected staff to support. Breaking away from the traditional forms of training, champions described accompaniment as an essential part of their role:

*‘My role is to encourage the staff and explain to them what it's about and encourage them to do it. If they don't understand, then to explain why we're doing it, the benefits,' (Participant 22, Nurse Champion).*

Another recalled, ‘I let them know that they can come at any time and ask me for help or who else they can talk to if they want more information…. I also told them who the other champions are that they can ask for help' (Participant 6, Nurse Champion).

Accompaniment also involved being a ‘sounding board' for other staff if they were uncertain about what to do in a particular case, or confronted by the challenges of responding to IPV presentations:

*‘A lot of them do have questions. They say, “Oh, I asked a patient the other day did she experience violence at home and she said yes and I didn't know what to do … So, did I do the right thing?” … I think it's very confronting for a lot of staff' (Participant 6, Nurse Champion).*

Champions' ongoing presence in the clinical setting allowed them to offer themselves for advice and support *‘…people can come to me and ask “can you help me with this screen?”' (Participant 2, Nurse Champion).*

Participants also reported using informal training as part of accompaniment to support staff in gaining confidence and to build screening into routine practice during the assessment process for each patient:

*‘I go, “oh well, what are you nervous about? Do you want me to help you? Do you want me to come with you? Have you got your little laminated card (with screening instructions) with you?” I just have this conversation and they go, “I'm nervous about this” and then they'll say, “Oh do you want to do it and I'll come with you?”' (Participant 6, Nurse Champion).*

Accompanying also involved ‘buddying' others to build confidence, capacity and ownership amongst clinicians (Participants 6, 8, 9 and 11). One champion spoke of working alongside clinicians:

‘*I ask them (ED staff) do they need any help? Ask them are they comfortable doing it? Would they like me to do it with them? Would they like me to do it for them and they could just observe? Ask them do they have any problems? Do they have any questions? Do they know what to do when they do identify someone who is a positive domestic violence victim?' (Participant 6, Nurse Champion).*

Practice change was also reinforced through constantly *‘monitoring screening and being proactive' (Participant 16, Manager Champion)* and *‘always being conscious of whether it's being done or not' (Participant 5, Nurse Champion)*. The majority of participants spoke about staff reminders as a form of accompaniment.

*‘[by constantly reminding] My aim is for people to see me and say, Oh, that's right I've got to do some screening' (Participant 2, Nurse Champion).*

#### 3.2.4. Serving as an Educator and Resource Person

Over time, champions became knowledgeable resource people within their units. They provided information and advice on the topic of IPV, along with preparing and providing resources and information for staff and patients. Describing how they operated as a resource person, a number spoke about their role in ‘*answering queries'* (*Participant 22, Nurse Champion*) and providing advice on cases and any issues as they arose. One participant referred to herself as a ‘liaison for staff' *(Participant 7, Nurse Champion)* for advice and suggestions or to escalate issues.

Participants identified the importance of building their own knowledge base and the importance of being viewed by staff as knowledgeable in order to be an effective resource person. Speaking of this, one participant recalled the need to *‘learn the process of how the IPV screening should be done… I had to expand my knowledge and then pass that on to the staff members' (Participant 6, Nurse Champion).* Another recalled increasing her knowledge and understanding and building her personal capability in order to become a credible resource for others:

*‘I went and met the girls next door at the Women's Health Centre and chatted to them and organised some in service. I went in there and asked them, “What do you offer?” ‘Cause I never knew. I rang the DV hotline and said to them, “I want to know what you offer, because I'm a nurse in ED and if I'm going to tell someone to ring your line I need to know what you can offer.” You know, just stuff like that so that you can then tell other staff' (Participant 22, Nurse Champion).*

This participant recounted developing a resource folder for the unit that staff could utilise to increase their understanding of IPV and their awareness of IPV services. At Site 2, Participant 3 recalled developing envelopes of information to give to patients who were identified through the screening process.

Most of the education and training champions provided staff was of an informal nature, and it was to supplement the formal training that each of the nursing staff received by the project lead prior to screening for IPV. Champions developed and utilised various tools to remind staff to screen. One participant spoke about a screening checklist board that she had developed that included the IPV screen. Another champion developed an IPV screening sticker which was a symbol, stuck on all the computers across the unit. The project officer champion worked with the electronic medical records team to place an icon reminder on the computer system. A number of participants spoke about utilising this system to remind staff to screen.

*‘I will flag people needing IPV screening with the FirstNet icon and then remind staff. Coz some people forget' (Participant 22, Nurse Champion).*

*‘I see it as my role to highlight the person who needs to have the screening as a prompt to other staff members because they're busy doing a lot of other things with a lot of patients and they just need a little reminder' (Participant 17, Nurse Champion).*

#### 3.2.5. Managing Resistance

Working around power dynamics was a key strategy for champions. Participants reported, *‘There's one or two Nurse Unit Managers that you have to work around' (Participant 6, Nurse Champion)* to ensure that the screening process continued. One participant spoke about there being a *‘big hierarchy in ED' (Participant 5, Nurse Champion)* and that being a relatively junior member of the staff, she *‘picked her battle'* by carefully choosing who she approached about the screening process. This sentiment was also shared by other participants:

*‘I pick the nurses that I [approach about screening]–like–some of the senior nurses that were there before me I find it very difficult… So, I wouldn't go- and some Nurse Unit Managers, some I would work around.' (Participant 6, Nurse Champion).*

*‘I wouldn't approach that nurse about anything. I might just wait till they're gone and sneak in and do the IPV assessment myself' (Participant 5, Nurse Champion).*

Many participants concentrated their training and accompaniment strategies on nursing students, junior nurses and nurses who were compliant-‘*You need to get them when they start' (Participant 5, Nurse Champion).*

Participants spoke about working around obstructions in a variety of ways. One shared, *‘If one thing doesn't work I'll try a different technique. So, I'll just keep going' (Participant 9, Nurse Champion).* Another shared, *‘quite often they (nurses) won't get up and do it' (the screening process). When I'm not busy I'll do a round of the department and go around and do all the IPV screens. I'll say, “There's a few IPV screens that haven't been done, do you want me to do them?” One or two nurses will go, “No no that's my job, I'll do it.” But some of them just go, “yeah you can do it.” Or some will go, “Oh look I want to do it but I've only done one and I feel a bit nervous”' (Participant 6, Nurse Champion).*

Some participants shared that their workaround was to ‘just take on the screening' (Participant 5, Nurse Champion) saying, ‘I will happily do an IPV screen on somebody else's patient… I think for me as a champion it's just part of my ED nursing now. Something I just do like wound care or whatever else that you do, it's just something that I do and I'm happy to. I understand that not everybody is as willing or as able or as comfortable to do it' (Participant 5, Nurse Champion).

## 4. Conclusion

The findings from the current study illuminate how champions can be employed to construct effective and meaningful change in response to IPV in the ED setting. Previous research has detailed the difficulties faced in bringing about change in practice and implementing IPV screening within EDs [10, 24, 41]. Health services are large traditional bureaucracies, where change can be difficult to implement. There are also power dynamics within health services based on traditions that must be complied with, for example, the tradition of ‘evidence-based practice' and what is seen as the traditional scope of practice for nurses within an ED.

Clinicians have reported that screening for IPV in the ED is both acceptable and appropriate [10]. Follow-up with women screened from a study in 2007 also found strong support for the screening process [42]. Although there is a body of knowledge examining barriers and facilitators to responding to IPV within EDs [10, 18], along with a significant body of knowledge about the use of champions in healthcare settings [18], there is a paucity of research regarding the champions of IPV practice change themselves [24]. Staff often feel unprepared and inadequately skilled to implement IPV screening [9] and any change in practice can be met with resistance.

Champions have been evaluated across a range of healthcare settings and contexts in areas such as speaking up behaviours [43], person centred care [44], health literacy [45] nutrition care [46], technology adoption in healthcare [47] and medication management and infection control [48]. Champion research has identified that, through sharing knowledge and expertise, clinical champions can promote evidence-based practice [26]. There is also moderate level systematic review evidence to confirm healthcare champions who function as local opinion leaders can improve compliance with evidence-based practice (Flodgren et al., 2019). In the main, the focus of past champion research has been on the effectiveness or attributes of champions. Characteristics include facilitating trust along with persistence, enthusiasm, being approachable [50] and motivating others and making sense of innovation [51].

Extending previous evidence about champions working within health systems, this study is the first to investigate in detail champions working to bring about IPV practice change in a healthcare setting. Our study highlights how nurse champions utilised transformative dialogue and relational connections, using storytelling of positive outcomes to build staff commitment and motivation. Champion's skill in the utilisation of narrative practice to socialise concepts and create a culture in which IPV could be openly spoken about with staff and patients alike were essential. Employing ethnographic case studies of telecare development Hendy and Barlow [51] noted that champions were highly effective when change was more localised, when championing was strongly identified with their work or contained within discrete aspects of practice. These findings resonate with our study where the immediate connection of championing to work allowed champions to narrate shared understandings about change that could be shared across their work team. This form of shared understanding is recognised to increase group cohesiveness and team identification [52].

Siemens proposed the learning theory of connectivism, which suggests that personal knowledge and learning occur through evolving networks within organisations, allowing learners to remain current in their field via connections within their network [53]. While previous research has identified the social influence of champions, the strategies employed by healthcare champions to achieve buy-in have received less attention [54]. Our findings illuminate how transformative dialogue, relationships, opposed realities and their related perceptions and practices can be transformed through the work of champions into shared understandings that inform change. Beyond understanding of culture and work practices, our findings suggest the embeddedness of clinical champions is vital to understanding custom and practice [55] which allows champions to navigate social relationships and hierarchy and overcome resistance.

Consistent with previous research [25, 26], the current study identified the importance and positive impact, of champions to quality improvement, practice change and addressing long-standing barriers. Unique outcomes from this study include rich detail on the multiple strategies that clinical champions utilised to enable practice change. The current study found that the seniority of champions assisted them to overcome resistance. Targeting junior staff who were at the beginning of their career to ensure that they were carrying out the screening process enabled champions to influence change within their units more easily.

Previous research has identified a number of structural issues that pose barriers to screening within EDs including competing demands, poor integration with medical records, lack of privacy and low level of staff knowledge and therefore confidence to screen [10, 18]. The findings of the current research suggest that champions can overcome structural barriers by working at the process level to bring about practice change. This finding reconceptualises the thinking about how to overcome structural issues that were previously thought to be barriers to screening for IPV within EDs. This form of clinical leadership can then be utilised to work around structural barriers and bring about sustained change in IPV routine screening practice within EDs.

Our findings have implications for continuing professional development across healthcare organisations. Even though champions were largely nurses, this study also included champions who were doctors, managers and executive leaders. The study demonstrates the importance of IPV training for clinicians and leaders, and how this training can impact the way that healthcare organisations respond to the needs of individuals who are experiencing IPV. Further, the translational implications of this study inform leadership and management training to incorporate learning about the role of champions, and how to work with champions effectively to bring about change and service improvement.

In accordance with previous evidence [8], this study found that training and support enabled staff to feel more confident and, over time, build screening into routine practice. Taking into consideration the complexities of IPV was vital. New skills were required for nurses to be able to deal with the complexities of IPV and with their own response to the suffering their patients experienced due to IPV. One of the previously reported characteristics of champions was to provide mentoring and expert advice [24, 56, 57]. The current study builds on this by providing evidence on the specific methods of training and accompaniment provided by champions and identifying the unique elements and sensitivity to the complexities of IPV required in order for training to be effective. The support champions provided was critical in this regard. Recently, Shea [54] highlighted that most healthcare research notes the presence or absence of champions and does not provide a better understanding of what makes champions more effective within specific settings or in relation to specific areas of practice [54]. The findings presented in this paper afford detailed insight into champions of IPV practice change. It offers valuable understanding for managers and those tasked with supporting change around how to better prepare, utilise and support champions.

## 5. Limitations

This study was conducted in two rural sites in NSW, Australia. This may limit the generalisability of the results to broader ED settings in other countries and in metropolitan areas. The study did not investigate the difference in impact between self-nominated champions and those who were nominated by their manager. Additionally, the study did not investigate the differences between male and female champions, or if there were any differences in strategies used by champions in relation to different forms of IPV. These topics warrant further research in the future. Finally, a more comprehensive analysis of the effect of champions is warranted. We hope that our study will provide the impetus for champions to be more rigorously evaluated in subsequent studies.

## 6. Implications for Nursing Practice

The findings offer new insight into how health services might better prepare and support champions. For nurse managers and health service managers, the findings may serve as a guide for supporting nurse champions who seek to make change in day-to-day practice. For those implementing change, the findings highlight the key things to consider when educating clinical change agents.

## Figures and Tables

**Table 1:** Participant characteristics (*n* = 23).

	Site one (*n* = 10) % (*n* =)	Site two (*n* = 11) % (*n* =)	Across health district (*n* = 2) % (*n* =)	Total (*n* = 23) % (*n* =)
*Gender*				
Female	70% (*n* = 7)	82% (*n* = 9)	100% (*n* = 2)	78% (*n* = 18)
Male	30% (*n* = 3)	18% (*n* = 2)		22% (*n* = 5)

*Work role*				
Frontline nurse	50% (*n* = 5)	64% (*n* = 7)		52% (*n* = 12)
Service level manager	30% (*n* = 3)	18% (*n* = 2)		22% (*n* = 5)
Senior clinical nurse (clinical nurse consultant, nurse educator)	10% (*n* = 1)	18% (*n* = 2)		13% (*n* = 3)
Executive leads			100% (*n* = 2)	9% (*n* = 2)
Director level manager	10% (*n* = 1)			4% (*n* = 1)

## Data Availability

The authors are unable to share the research data as ethics approval was not granted for this.
